# Vitrification with shortened equilibration across human embryonic developmental stages: a proof-of-concept study

**DOI:** 10.1093/hropen/hoag053

**Published:** 2026-06-03

**Authors:** Noya Doolman, Eden Weinberg, Ettie Maman, Shmuel Inbar, Moran Shapira, Raoul Orvieto, Adva Aizer

**Affiliations:** Gray Faculty of Medical and Health Sciences, Tel Aviv University, Tel Aviv, Israel; Infertility and IVF Institute, Department of Obstetrics and Gynecology, Sheba Medical Center, Ramat Gan, Israel; Academy of Clinical Embryology, Sheba Medical Center, Ramat Gan, Israel; Gray Faculty of Medical and Health Sciences, Tel Aviv University, Tel Aviv, Israel; Infertility and IVF Institute, Department of Obstetrics and Gynecology, Sheba Medical Center, Ramat Gan, Israel; Infertility and IVF Institute, Department of Obstetrics and Gynecology, Sheba Medical Center, Ramat Gan, Israel; Gray Faculty of Medical and Health Sciences, Tel Aviv University, Tel Aviv, Israel; Infertility and IVF Institute, Department of Obstetrics and Gynecology, Sheba Medical Center, Ramat Gan, Israel; Gray Faculty of Medical and Health Sciences, Tel Aviv University, Tel Aviv, Israel; Infertility and IVF Institute, Department of Obstetrics and Gynecology, Sheba Medical Center, Ramat Gan, Israel; Academy of Clinical Embryology, Sheba Medical Center, Ramat Gan, Israel; Gray Faculty of Medical and Health Sciences, Tel Aviv University, Tel Aviv, Israel; Infertility and IVF Institute, Department of Obstetrics and Gynecology, Sheba Medical Center, Ramat Gan, Israel; Academy of Clinical Embryology, Sheba Medical Center, Ramat Gan, Israel

**Keywords:** shortened equilibration vitrification, blastocyst re-expansion, one-step warming, blastocyst vitrification, microdrops vitrification, 3PN zygotes, ultra-fast vitrification, ultra-fast warming

## Abstract

**STUDY QUESTION:**

Can vitrification with a shortened equilibration solution (ES) be safely applied to human embryos without compromising key embryological clinical outcomes?

**SUMMARY ANSWER:**

Vitrification with shortened equilibration was associated with comparable observed survival, developmental progression, and blastocyst re-expansion rates when compared to standard vitrification.

**WHAT IS KNOWN ALREADY:**

Vitrification with shortened-equilibration exposure has been primarily evaluated in mouse models and in human oocytes at the germinal vesicle and metaphase II stages, where short exposure times have been shown to maintain post-warming survival and preserve mitochondrial integrity comparable to conventional vitrification. The European Commission (CE) approval exists for the use of shortened-equilibration vitrification protocols in human oocytes, although clinical experience remains limited. Conversely, evidence regarding the application of shortened-equilibration vitrification to human embryos is currently lacking, and there is currently no clinical regulatory approval for embryos or blastocyst vitrification using shortened-equilibration protocols.

**STUDY DESIGN, SIZE, DURATION:**

Prospective proof-of-concept study performed in a single IVF laboratory from April 2025 to March 2026. It involved 45 human blastocysts, 49 cleavage-stage embryos, and 102 tripronuclear (3PN) zygotes. Blastocysts were allocated to standard, shortened-equilibration vitrification using full-volume (300 µl) or microdrop loading (50 µl) vitrification. 3PN zygotes were used as a model for early-stage embryo response and were vitrified immediately after fertilization or at the cleavage stage according to group allocation, using standard and shortened-equilibration protocols.

**PARTICIPANTS/MATERIALS, SETTING, METHODS:**

All blastocysts were identified as affected by preimplantation genetic testing for monogenic disorders (PGT-M) and used for research only; they were cryopreserved, then warmed using standard protocols, then re-vitrified per group using shortened-equilibration vitrification and subsequently warmed using one-step warming. 3PN zygotes were included as clinically relevant research material and assigned to standard vitrification or shortened-equilibration vitrification (300 µl, 2 min; microdrops 50 µl, 2–3 min). Outcomes included survival rate, day-3 development, blastocyst formation, iDAScore (morphokinetic assessment), and post-warming re-expansion.

**MAIN RESULTS AND THE ROLE OF CHANCE:**

3PN zygotes were allocated to four groups: standard vitrification and shortened-equilibration vitrification using full-volume loading (300 µl, 2-min) or microdrop loading (50 µl, 2 or 3-min). Baseline characteristics were comparable between groups. Post-warming survival rates ranged from 89.2% to 100% (*P* = 0.408). Day-3 development ranged from 47% to 60% (*P* = 0.868). Blastocyst formation ranged from 32.0% to 41.2% (*P* = 0.920). The mean iDAScore ranged from 2.25 to 4.07 (*P* = 0.281). In cleavage-stage embryos, survival ranged from 78.6% to 95.2% (*P* = 0.325), and blastocyst formation from 25.0% to 55% (*P* = 0.262). In blastocysts, survival ranged from 93.3% to 100% (*P* = 0.592). Re-expansion at 2 h ranged from 82% to 90% after first warming (*P* = 0.156) and from 82% to 85% after second warming (*P* = 0.784). Time to complete re-expansion ranged from 3.6 to 4.0 h (*P* = 0.893).

**LIMITATIONS, REASONS FOR CAUTION:**

The sample sizes were limited, and the study may be underpowered to detect differences between groups; patient distribution was unbalanced due to random allocation of samples between groups. In addition, the use of 3PN zygotes limits direct comparison with established blastocyst quality benchmarks due to their inherently reduced developmental potential.

**WIDER IMPLICATIONS OF THE FINDINGS:**

Shortened-equilibration vitrification demonstrated comparable post-warming survival, developmental progression, and blastocyst re-expansion to standard vitrification. These findings support the feasibility of shortening equilibration time without compromising key embryological outcomes. If validated in larger studies, this approach may contribute to optimizing and simplifying vitrification protocols in clinical practice.

**FUNDING:**

This research received no specific external grant from funding agencies in the public, commercial, or not-for-profit sectors. Departmental and institutional resources from the Infertility and IVF Institute, Sheba Medical Center, supported the study and manuscript preparation. The laboratory also receives general research scholarship support from the Alrov Fund.

**DISCLOSURES:**

R.O. reports speaker's fees from Merck Group. The other authors declare that they have no competing interests.

**TRIAL REGISTRATION NUMBER:**

N/A

WHAT DOES THIS MEAN FOR PATIENTS? Freezing and warming embryos are essential steps in IVF treatment, but standard methods involve several handling steps and exposure to special solutions. In this study, we examined whether a shorter preparation step before embryo freezing, together with a simplified warming approach, could be used safely. We found that embryos processed using these simplified methods showed similar survival and early development compared with standard methods. These findings suggest that simplifying embryo freezing and warming procedures may be safe, although further studies are needed before routine clinical use.

## Introduction

Cryopreservation of oocytes and embryos is an integral component of modern ART, enabling fertility preservation, flexible treatment scheduling, preimplantation genetic testing (PGT), and the widespread adoption of freeze-all strategies. The increasing use of frozen embryo transfer (FET) has been associated with improved cumulative live birth rates while enhancing treatment safety, through higher rates of single embryo transfer and a reduced risk of ovarian hyperstimulation syndrome (OHSS) (Griesinger *et al.*, 2011; Maheshwari *et al.*, 2018; Li *et al.*, 2019; Smeenk *et al.*, 2023; Orvieto, 2025). In parallel, advances in extended embryo culture to the blastocyst stage and the integration of genetic testing have strengthened embryo selection and optimized the clinical use of cryopreservation in contemporary IVF practice (Gardner *et al.*, 1998; Blake *et al.*, 2007; Penzias *et al.*, 2018; Practice Committees of the American Society for Reproductive Medicine and the Society for Assisted Reproductive Technology, 2024).

The concept of vitrification was introduced in the mid-1980s as an alternative to controlled-rate slow freezing, aiming to prevent intracellular ice formation by inducing a glass-like state during cooling (Fahy *et al.*, 1984; Rall and Fahy, 1985). While early applications demonstrated feasibility, clinical use was initially limited by insufficient cooling and warming rates. Subsequent advances in carrier systems that minimized solution volume, including minimal drop vitrification (Arav, 1992), the hemistraw system (Vanderzwalmen *et al.*, 2002), and open carriers (Kuwayama *et al.*, 2005; Cobo *et al.*, 2008), enabled rates compatible with routine clinical practice and established vitrification as the standard method for oocyte and embryo cryopreservation.

From a biological and biophysical perspective, successful vitrification relies on rapid cellular dehydration combined with high concentrations of cryoprotective agents (CPA) to prevent ice nucleation and growth. Exposure to an equilibration solution induces osmotic shrinkage, as water exits the cell faster than CPA permeates the plasma membrane, followed by gradual CPA entry and partial volume recovery as equilibration progresses (Mazur, 1963; Rall, 1987). Achieving an optimal balance between sufficient dehydration and avoidance of excessive osmotic stress or CPA-related toxicity is therefore critical for cell survival and subsequent developmental competence.

Despite technological progress, equilibration protocols used prior to vitrification have remained largely consistent over time. Most clinical protocols rely on equilibration steps of 5–15 min prior to cooling, adjusted according to cell type and developmental stage (Kuwayama *et al.*, 2005; Cobo *et al.*, 2010; Rienzi *et al.*, 2010). These durations were established empirically to support cell survival and are still widely used across oocytes, cleavage-stage embryos, and blastocysts. From a practical perspective, prolonged equilibration may introduce limitations, including increased handling time and extended exposure to cryoprotectants.

Recent experimental and modelling studies have challenged the need for prolonged equilibration during vitrification. Computational analyses indicated that human oocytes reach minimal volume and intracellular solute concentrations within 30–60 s of exposure to equilibration solution, with predicted ice formation risk comparable to that after standard equilibration durations (Gallardo *et al.*, 2019). Consistent with these findings, proof-of-concept studies in human oocytes demonstrated high post-warming survival following markedly shortened equilibration protocols, supporting the feasibility of rapid vitrification preparation that reduced equilibration exposure during vitrification (Schiewe *et al.*, 2024; Wozniak *et al.*, 2024). Complementary animal data suggest potential differences in cellular ultrastructure preservation, including mitochondrial organization and endoplasmic reticulum integrity, following reduced equilibration vitrification approaches (Cho *et al.*, 2024).

In parallel, increasing attention has focused on simplifying and shortening post-warming procedures. Warming protocols using one-step rehydration have shown survival and clinical outcomes comparable with, and in some studies improved, conventional multi-step approaches in large human cohorts, while substantially reducing handling time and exposure outside the incubator (Liebermann *et al.*, 2024). These protocols have been successfully implemented in clinical practice for both oocytes and embryos, supporting the safety and efficiency of reducing procedural complexity during warming.

Despite the increasing clinical use of one-step warming protocols, evidence supporting shortened-equilibration vitrification, specifically shortening the equilibration step prior to cooling, remains limited. For oocytes, regulatory approval exists, and a small number of human studies suggest that shortened-equilibration vitrification combined with one-step rehydration can maintain high post-warming survival and support subsequent development (Schiewe *et al.*, 2024; Wozniak *et al.*, 2024). However, clinical data are still scarce. For embryos, clinical evidence is currently lacking. Importantly, while most available evidence focuses on oocytes, the applicability of shortened-equilibration vitrification to more complex multicellular structures such as cleavage-stage embryos and blastocysts remains largely unexplored. These developmental stages differ in membrane permeability, cell-to-cell interactions, and sensitivity to osmotic stress, which may influence their response to shortened equilibration protocols. As a result, whether vitrification protocols can be safely shortened without compromising embryological outcomes remains an important knowledge gap.

The present proof-of-concept study, therefore, aimed to assess the safety of shortened-equilibration vitrification with a markedly shortened equilibration step in human zygotes and embryos, including post-warming survival, early developmental progression of zygotes and cleavage-stage embryos, and blastocyst re-expansion as primary endpoints.

## Materials and methods

### Study population and evaluation

We conducted a prospective observational proof-of-concept study including 102 tripronuclear (3PN) zygotes from 57 patients, 49 cleavage-stage embryos from 29 patients, and 45 blastocysts from 13 patients, treated between April 2025 and March 2026 at Sheba Medical Center. Patients were allocated to the study groups using a computer-based randomization tool (Randomizer, Ghostware, Switzerland). For 3PN zygotes and cleavage-stage embryos, all corresponding samples were assigned according to the patient’s group allocation. In contrast, in the blastocyst cohort, allocation was performed at the embryo level, allowing embryos from the same patient to be assigned to different vitrification protocols. Study groups included standard vitrification and shortened-equilibration vitrification protocols using either full-volume loading (300 µl) or microdrop loading (50 µl).

### Ethical approval

The study was approved by the Institutional Review Board (Helsinki Committee) of Sheba Medical Center (approval no. 0187-23-SMC) and was conducted in accordance with the Declaration of Helsinki. All patients provided written informed consent for participation.

### Vitrification and warming protocols

**Figure 1.**
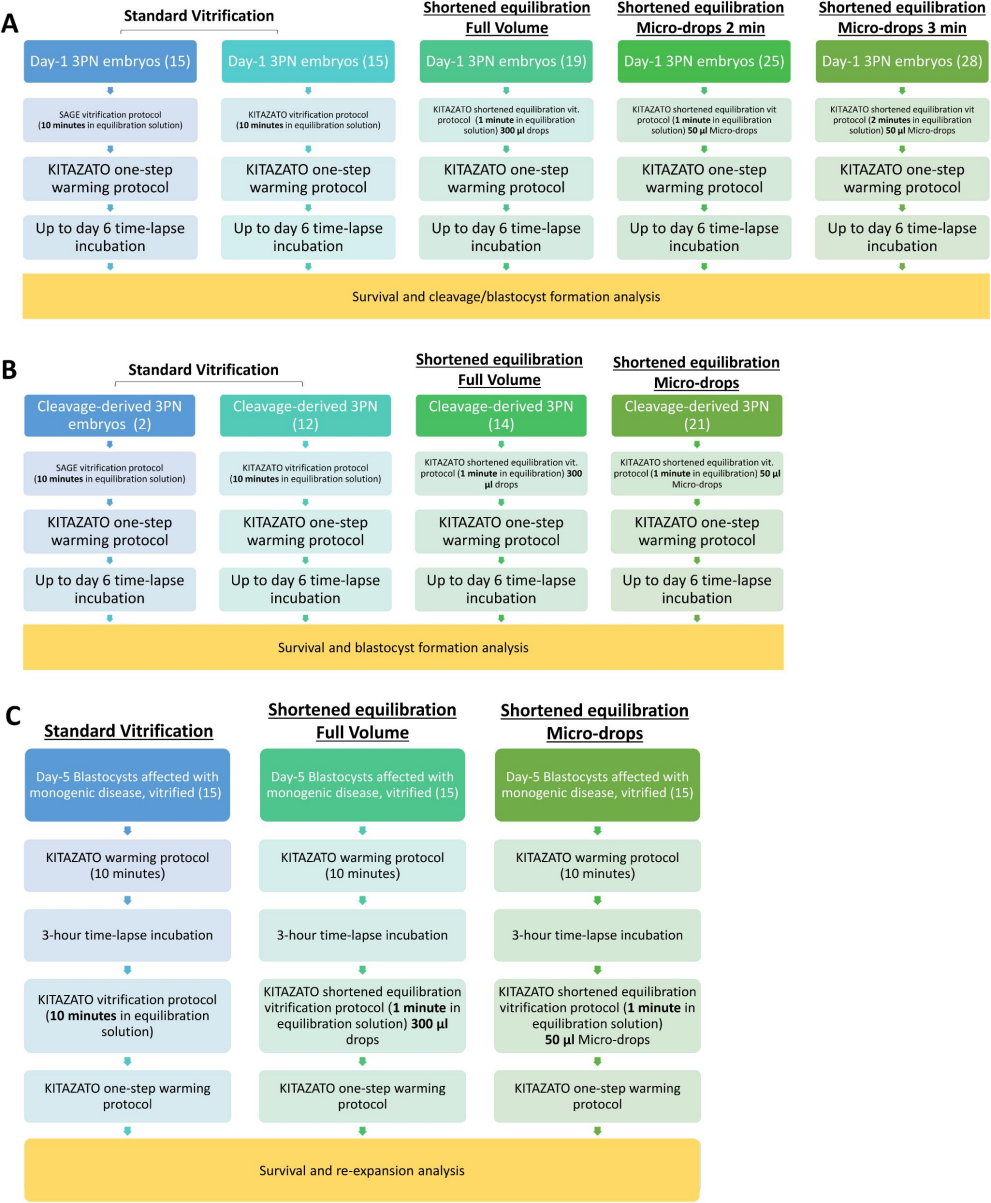
**Study design and sample allocation according to vitrification protocol**. 3PN zygotes, cleavage-stage embryos, and blastocysts were allocated to standard vitrification or shortened-equilibration vitrification using full-volume (300 µl) or microdrop (50 µl) loading. (**A**) Zygotes were vitrified after fertilization assessment. (**B**) Cleavage-stage embryos were vitrified on days 2–3. (**C**) Blastocysts underwent standard vitrification and warming followed by re-vitrification and one-step warming. 3PN, tripronuclear; PGT-M, preimplantation genetic testing for monogenic disorders.

All vitrification and warming procedures are summarized in the study flowchart (Fig. 1). 3PN zygotes were included in the study. During the study period, both Sage (CooperSurgical, Måløv, Denmark) and Kitazato (Kitazato, Shizuoka, Japan) media systems were used for conventional vitrification due to a transition in laboratory practice. Zygotes were vitrified immediately following fertilization assessment and allocation to study groups. They were assigned to one of the following vitrification protocols: standard vitrification, shortened-equilibration vitrification using a full-volume loading method (300 µl), or shortened-equilibration vitrification using microdrop loading (50 µl). For the microdrop loading protocol (50 µl), equilibration incubation was performed for either 2 or 1 min followed by approximately 60 s exposure to vitrification solution prior to loading and cooling, resulting in total pre-cooling exposure times of approximately 3 and 2 min, respectively. All 3PN zygotes were subsequently warmed using the one-step warming protocol (Fig. 1A).

Cleavage-stage embryos derived from 3PN fertilization were vitrified on day 2 or day 3 following ICSI. Vitrification timing was determined according to routine laboratory workflow. In accordance with patient-level group allocation, embryos were assigned to one of three vitrification protocols: standard vitrification, shortened-equilibration vitrification using full-volume loading (300 µl), or shortened-equilibration vitrification using microdrop loading (50 µl) with a 1-min equilibration incubation (Fig. 1B). All cleavage-stage embryos were subsequently warmed using the same one-step warming protocol.

All blastocysts included in the study were identified as affected following preimplantation genetic testing for monogenic disorders (PGT-M) and were used exclusively for research purposes. All blastocysts were initially vitrified using the Sage system as part of routine clinical practice in our laboratory, ensuring uniform baseline conditions across study groups. Blastocysts were derived from PGT-M cycles and were not routinely assessed for ploidy status. Blastocysts were initially cryopreserved and warmed using a standard vitrification and warming protocol, followed by re-vitrification according to group allocation using either standard or shortened-equilibration vitrification protocols. Subsequent warming was performed using a one-step warming protocol (Fig. 1C).

Standard vitrification was performed using vitrification kits from Sage or Kitazato. Both vitrification systems are based on standard cryoprotectant formulations. The equilibration solution typically contains approximately 7.5% ethylene glycol and 7.5% dimethyl sulfoxide, while the vitrification solution contains approximately 15% of each permeating cryoprotectant, supplemented with a non-permeating agent such as sucrose, according to the manufacturers’ specifications. Shortened-equilibration vitrification and all warming procedures were performed using Kitazato media (Kitazato). Standard warming was applied only for the first warming of blastocysts (Fig. 1C) and consisted of 1 min in 300 µl warming solution (TS) pre-warmed to 37°C, followed by 3 min in 100 µl dilution solution (DS) at room temperature, and 5 min plus an additional 1 min in washing solution (WS) at room temperature. DS and WS droplets were overlaid with Sage oil (CooperSurgical).

One-step warming was performed according to the manufacturer’s instructions (Kitazato) and consisted of 1 min in 1000 µl warming solution, containing 1.2 M trehalose according to the manufacturer’s specifications, using pre-warmed media and culture dishes maintained at 37°C, followed by three washes in culture medium prior to loading into EmbryoSlide dishes.

For vitrification of zygotes and cleavage-stage embryos derived from 3PN fertilization, Cryotop™ devices were used (Kitazato, Japan). Blastocysts were vitrified using either Cryotop™ or VitriFit™ devices (Kitazato or CooperSurgical), selected randomly according to routine clinical laboratory practice.

### Embryo culture and time-lapse monitoring

Following warming, all zygotes and embryos were cultured in 1-Step SPS medium (Origio, CooperSurgical) using the EmbryoScope™ time-lapse incubation system (Unisense FertiliTech, Vitrolife, Aarhus, Denmark) until day 6 of development or re-vitrification.

Outcome measures were assessed according to group relevance and included post-warming survival rate, early developmental progression, day-3 development, blastocyst formation, morphokinetic assessment using the iDAScore, and post-warming blastocyst re-expansion.

Blastocyst re-expansion was evaluated 2 h post-warming using time-lapse images. Images were exported and analyzed with GeoGebra graphic calculator software (GeoGebra GmbH, Linz, Austria). The polygon tool was used to manually trace the external boundary of the re-expanded blastocyst. In parallel, the inner boundary of the zona pellucida was traced, representing the original blastocyst size prior to vitrification. The re-expansion rate was calculated as the ratio between the blastocyst area and the inner zona pellucida area (Supplementary Fig. S1). This method was applied uniformly across all analyzed blastocysts.

### Statistical analysis

Continuous variables were assessed for normality. Normally distributed variables were compared using one-way ANOVA with Bonferroni post hoc correction, while non-normally distributed variables were analyzed using the Kruskal–Wallis test. Categorical variables were compared using the chi-square test or Fisher’s exact test, as appropriate. Significance was accepted at *P* < 0.05. Given the proof-of-concept design and limited sample size, analyses were considered exploratory. Statistical analyses were conducted using IBM SPSS v.23 (IBM Corporation Inc, Chicago, IL, USA).

## Results

The clinical and stimulation characteristics of patients are presented in Tables 1, 2, and 3. Overall, baseline characteristics were comparable between vitrification groups across all study arms, including zygote-stage, cleavage-stage, and blastocyst-stage analyses. No significant differences were observed in maternal age, BMI, ovarian response parameters, hormonal levels, or stimulation protocols.

**Table 1.** Baseline patient and cycle characteristics in the zygote-stage vitrification cohort.

	Standard vitrification	Shortened-equilibration vitrification	Shortened-equilibration vitrification	Shortened-equilibration vitrification	*P*-value
		300 µl, 2 min	50 µl, 2 min	50 µl, 3 min	
No. of patients	24	16	21	13	
No. of 3PN zygotes	30	19	25	28	
OS characteristics					
Mean oocyte retrieved/OPU (mean ± SD)	13.75** ± **8.9	11.94** ± **7.1	14.1** ± **7.5	15.69** ± **6.8	0.655
2PN fertilization/MII (%)	176/279 (63.1)	118/169 (69.8)	159/239 (66.5)	98/163 (60.1)	0.250
3PN fertilization/oocyte retrieved (%)	33/330 (10)*	21/191 (10.9)*	25/296 (8.4)*	33/204 (16.2)	0.047
Mean peak estradiol levels pmol/l (mean ± SD)	9018** ± **7782	7120** ± **3912	11 874** ± **7573	12 427** ± **7824	0.186
Mean peak progesterone levels nmol/l (mean ± SD)	2.84** ± **1.9	2.95** ± **2	3.3** ± **1.9	5.9** ± **8.8	0.167
Antagonist protocol (%)	22/24 (91.7)	15/16 (93.7)	20/21 (95.2)	13/13 (100)	0.756
Age, years (mean ± SD)	38.54** ± **5.1	37** ± **4.8	34.57** ± **5.6	34.38** ± **3.9	0.656
BMI, kg/m^2^ (mean ± SD)	22.43** ± **3.3	24.87** ± **5.4	25.28** ± **6.5	25.6** ± **7.9	0.409
Smoking (%)	4/24 (16.7)	6/16 (37.5)	5/21 (23.8)	3/13 (23.1)	0.515

Data are presented as mean ± SD or n (%). Statistical analysis was performed using one-way ANOVA for continuous variables and chi-square or Fisher’s exact test for categorical variables, as appropriate.

* *P* < 0.05.

OPU, oocyte pickup; MII, metaphase II; 2PN, two pronuclei; 3PN, tripronuclear.

**Table 2.** Baseline patient and cycle characteristics in the cleavage-stage vitrification cohort.

	Standard vitrification	Shortened-equilibration vitrification	Shortened-equilibration vitrification	*P*-value
		300 µl, 2 min	50 µl, 2 min	
No. of patients	8	11	10	
No. of 3PN zygotes	14	14	21	
OS characteristics				
Mean oocyte retrieved/OPU (mean ± SD)	12.25** ± **6.6	15.36** ± **8.3	13.6** ± **5.7	0.662
2PN fertilization/MII (%)	47/84 (55.9)*	107/146 (73.2)*	73/120 (60.8)*	0.015
3PN fertilization/oocyte retrieved (%)	14/98 (14.3)	14/169 (8.2)	21/136 (15.4)	0.102
Mean peak estradiol levels pmol/l (mean ± SD)	9110** ± **7216	13 586** ± **9386	8115** ± **6283	0.291
Mean peak progesterone levels nmol/l (mean ± SD)	4.01** ± **1.9	4.09** ± **2	2.94** ± **1.5	0.343
Antagonist protocol (%)	7/8 (87.5)	11/11 (100)	8/10 (80)	0.282
Age, years (mean ± SD)	36.62** ± **5	34.91** ± **6.4	38.5** ± **3.4	0.335
BMI, kg/m^2^ (mean ± SD)	23.07** ± **5.7	22** ± **4.5	23.03** ± **2	0.873
Smoking (%)	1/8 (12.5)	2/11 (18.1)	1/10 (10)	0.103

Data are presented as mean ± SD or n (%). Statistical analysis was performed using one-way ANOVA for continuous variables and chi-square or Fisher’s exact test for categorical variables, as appropriate.

* *P* < 0.05.

OPU, oocyte pickup; MII, metaphase II; 2PN, two pronuclei; 3PN, tripronuclear.

**Table 3.** Baseline patient and cycle characteristics in the blastocyst vitrification cohort.

	Standard vitrification	Shortened-equilibration vitrification	Shortened-equilibration vitrification	*P*-value
		300 µl, 2 min	50 µl, 2 min	
No. of patients	6	10	6	
No. of blastocysts	15	15	15	
OS characteristics				
Mean oocyte retrieved/OPU (mean ± SD)	22.83 ± 6.5	28.4 ± 16.8	29.3 ± 15.1	0.239
2PN fertilization/oocyte retrieved (%)	99/137 (72.3)	213/284 (81.3)	176/230 (76.5)	0.660
Mean peak estradiol levels pmol/l (mean ± SD)	12 482 ± 6702	16 814 ± 16 868	22 354 ± 19 329	0.601
Mean peak progesterone levels nmol/l (mean ± SD)	2.3 ± 0.6	3 ± 1.4	3.1 ± 1.6	0.529
Antagonist protocol (%)	6/6 (100)	9/10 (90)	6/6 (100)	0.533
Age, years (mean ± SD)	33 ± 3.5	33.78 ± 3.6	32.96 ± 2.8	0.874
BMI, kg/m^2^ (mean ± SD)	22 ± 3.2	25 ± 6.2	26 ± 6.3	0.488
Smoking (%)	1/6 (16.7)	3/10 (30)	2/6 (33.3)	0.149
Blastocyst characteristics				
High-quality blastocysts (Gardner grade ≥ AA/AB/BA) (%)	8/15 (53%)	12/15 (80%)	9/15 (60%)	0.283
Pre-vitrification iDAScore (mean ± SD)	6.74 ± 2.3	7.91 ± 1.4	6.33 ± 2.3	0.179
Re-expansion degree in %, 2 h post-warming. First warming. (mean ± SD)	82% ± 12%	90% ± 11%	89% ± 12%	0.156

Data are presented as mean ± SD or n (%). Statistical analysis was performed using one-way ANOVA for continuous variables and chi-square or Fisher’s exact test for categorical variables, as appropriate.

* *P* < 0.05.

OPU, oocyte pickup; 2PN, two pronuclei.

In the zygote-stage cohort (Table 1), a significant difference was observed in the proportion of 3PN fertilization relative to oocytes retrieved (*P* = 0.047), with the highest rate in the shortened-equilibration vitrification microdrop 3-min group. In the cleavage-stage cohort (Table 2), a significant difference was observed in the 2PN fertilization rate (*P* = 0.015), with higher rates in the shortened-equilibration vitrification 300 µl group. No other significant differences were identified. No significant differences in baseline characteristics were observed in the blastocyst cohort (Table 3).

### Post-warming developmental competence of 3PN zygotes

Post-warming survival rates of 3PN zygotes were high and comparable across vitrification protocols, ranging from 89.2% to 100% (*P* = 0.408) (Table 4).

**Table 4.** Post-warming developmental competence of 3PN embryos according to vitrification protocol.

	Standard vitrification	Shortened-equilibration vitrification	Shortened-equilibration vitrification	Shortened-equilibration vitrification	*P*-value
		300 µl, 2 min	50 µl, 2 min	50 µl, 3 min	
No. of 3PN zygotes	30	19	25	28	
Survival rate (%)	28/30 (93.3)	17/19 (89.5)	25/25 (100)	25/28 (89.2)	0.408
Top-intermediate day 3/survived embryos (%)	16/28 (57.1)	8/17 (47)	15/25 (60)	14/25 (56)	0.868
No. of blastocyst/survived embryos (%)	9/28 (32.1)	7/17 (41.2)	8/25 (32)	9/25 (36)	0.920
Day 5 A/B Grade embryos/survived embryos (%)	2/28 (7.1)	1/17 (5.9)	2/25 (8)	2/25 (8)	
iDAScore (mean ± SD)	4.07 **± **2	2.25 **± **1.13	3.05 **± **1.9	3.31** ± **1.57	0.281

Data are presented as mean ± SD or n (%). Statistical analysis was performed using one-way ANOVA for continuous variables and chi-square or Fisher’s exact test for categorical variables, as appropriate.

3PN, tripronuclear.

The proportion of top- to intermediate-quality embryos on day 3 was similar between groups, ranging from 47% to 60% (*P* = 0.868). Blastocyst formation rates per survived embryos were also comparable across groups, ranging from 32.0% to 41.2% (*P* = 0.920) (Table 4).

Morphokinetic assessment using iDAScore for blastocysts ranged from 2.25 to 4.07, with no statistically significant differences between groups (*P* = 0.281).

### Development following cleavage-stage vitrification

Survival rates following cleavage-stage vitrification were comparable between groups, ranging from 78.6% to 95.2% (*P* = 0.325) (Table 5).

**Table 5.** Post-warming developmental competence of cleavage-stage vitrified 3PN-derived embryos according to vitrification protocol.

	Standard vitrification	Shortened-equilibration vitrification	Shortened-equilibration vitrification	*P*-value
		300 µl, 2 min	50 µl, 2 min	
No. of 3PN cleavage-stage embryos	14	14	21	
Survival rate (%)	12/14 (85.7)	11/14 (78.6)	20/21 (95.2)	0.325
No. of blastocyst/survived embryos (%)	3/12 (25)	4/11 (36.3)	11/20 (55)	0.262

Data are presented as n (%). Statistical analysis was performed using chi-square or Fisher’s exact test, as appropriate.

3PN, tripronuclear.

Blastocyst formation rates per survived embryos ranged from 25% to 55%, without statistically significant differences between groups (*P* = 0.262), although a numerical difference was observed (Table 5).

### Blastocyst survival and re-expansion kinetics

Post-warming survival rates of vitrified blastocysts were high and comparable between groups, ranging from 93.3% to 100% (*P* = 0.592) (Table 6). Data from the initial warming following standard vitrification are presented separately as part of the baseline characteristics (Table 3).

**Table 6.** Post-warming survival and re-expansion kinetics of vitrified blastocysts according to vitrification protocol.

	Standard vitrification	Shortened-equilibration vitrification	Shortened-equilibration vitrification	*P*-value
		300 µl, 2 min	50 µl, 2 min	
No. of patients	6	10	6	
No. of blastocysts	15	15	15	
Survival rate (%)	14/15 (93.3)	14/15 (93.3)	15/15 (100)	0.592
Re-expansion degree in %, 2 h post-warming. Second warming (mean ± SD)	82% **± **29%	84% **± **13%	85% **± **14%	0.784
Time to 100% re-expansion recovery in hours (mean ± SD)	3.6** ± **2.44	3.7 **± **2.1	4 **± **2.2	0.893

Data are presented as mean ± SD or n (%). Statistical analysis was performed using one-way ANOVA for continuous variables and chi-square or Fisher’s exact test for categorical variables, as appropriate.

Following re-vitrification and a second warming using the one-step warming protocol, re-expansion at 2 h ranged from 82% to 85%, with no statistically significant differences between groups (*P* = 0.784) (Table 6). Time to complete re-expansion following the second warming ranged from 3.6 to 4.0 h (*P* = 0.893).

## Discussion

This proof-of-concept study demonstrates that shortened-equilibration vitrification with a markedly shortened equilibration step does not compromise post-warming survival or early developmental competence across different developmental stages. Comparable outcomes were observed in zygotes, cleavage-stage embryos, and blastocysts, including survival rates, progression to blastocyst, and post-warming re-expansion. These findings suggest that reducing equilibration duration may be feasible without adversely affecting key embryological parameters, supporting the potential for simplifying vitrification protocols while maintaining biological integrity.

Blastocyst formation rates observed in the present study were lower than those typically reported in clinical IVF settings, where higher developmental competence is expected according to the Vienna consensus criteria (ESHRE Special Interest Group of Embryology, 2017). This finding is consistent with the use of 3PN zygotes, which are known to exhibit reduced developmental potential, with reported blastulation rates of approximately 10–30% in previous studies (Uzun *et al.*, 2021). The limited developmental capacity of 3PN embryos is primarily attributed to underlying chromosomal abnormalities, including triploidy and aneuploidy, which frequently result in developmental arrest prior to or during the blastocyst stage (Uzun *et al.*, 2021). Therefore, the observed blastocyst formation rates should be interpreted within this biological context rather than as an indicator of vitrification efficiency. Accordingly, a standard vitrification control group was included to allow internal comparison and to better isolate the effect of the vitrification protocol itself.

The findings of this study are consistent with the biophysical principles underlying vitrification. Cellular dehydration and cryoprotectant permeation occur rapidly following exposure to equilibration solutions, with previous modelling studies suggesting that critical intracellular conditions may be reached within a short time frame (Mazur, 1963; Rall, 1987; Gallardo *et al.*, 2019). Therefore, prolonged equilibration may not be essential to achieve adequate dehydration prior to cooling.

In cleavage-stage embryos, blastocyst formation rates following vitrification differed numerically between groups, with rates of 25.0% in the standard vitrification group and 36.3% in the shortened-equilibration vitrification full-volume group compared with 55% in the shortened-equilibration vitrification microdrop group. Although this difference did not reach statistical significance, likely due to the limited sample size, the magnitude of the observed difference suggests that it should be interpreted with caution rather than considered negligible. These findings should therefore be considered exploratory and require validation in larger cohorts.

Furthermore, reducing exposure duration may limit cumulative toxicity associated with cryoprotective agents and decrease osmotic stress, both of which are known to affect cell survival and developmental potential (Mazur, 1963). Together, these mechanisms may explain why shortening the equilibration step did not negatively impact post-warming outcomes across the different developmental stages evaluated in this study.

The present findings are in line with recent studies evaluating shortened-equilibration vitrification in human oocytes. Previous reports have shown that markedly shortened equilibration steps, combined with one-step warming approaches, can achieve high post-warming survival rates and support subsequent developmental potential (Schiewe *et al.*, 2024; Wozniak *et al.*, 2024). In addition, computational modelling has suggested that the intracellular conditions required for successful vitrification may be reached within a short exposure time, further supporting the feasibility of reduced equilibration durations (Gallardo *et al.*, 2019). Importantly, while these studies have focused primarily on oocytes, the current results extend these observations to embryonic stages, demonstrating comparable outcomes following shortened-equilibration vitrification across zygotes, cleavage-stage embryos, and blastocysts.

While most available evidence on shortened-equilibration vitrification has focused on oocytes, its applicability to embryonic stages has remained insufficiently explored. In contrast to earlier developmental stages, blastocysts contain a fluid-filled cavity, which may influence their response to vitrification and increase sensitivity to osmotic shifts during cryopreservation. In the present study, comparable post-warming survival and developmental outcomes were observed across zygotes, cleavage-stage embryos, and blastocysts, supporting the feasibility of extending shortened-equilibration vitrification to embryonic stages. Notably, blastocysts had undergone prior biopsy, which may partially mimic the effect of artificial collapse. However, no additional artificial collapse was performed before shortened-equilibration vitrification following the second warming. Despite this, favorable outcomes were maintained.

In a clinical setting, artificial collapse may still be considered to facilitate fluid exchange and potentially optimize vitrification efficiency. Previous studies have shown that artificial collapse prior to vitrification may improve post-warming survival rates, although without a consistent impact on implantation outcomes (Van Landuyt *et al.*, 2015). Other reports have demonstrated faster re-expansion kinetics in artificially collapsed blastocysts following warming (Kovačič *et al.*, 2018). In addition, some clinical studies have suggested improved implantation and pregnancy rates following artificial collapse prior to vitrification (Darwish and Magdi *et al.*, 2016).

From a clinical perspective, shortening the equilibration step may offer practical advantages in routine IVF laboratory workflows. Reduced handling time and shorter exposure to cryoprotective agents may improve efficiency and decrease the overall time embryos remain outside controlled culture conditions.

In addition, simplified vitrification protocols may contribute to improved standardization and reduce operator-dependent variability, particularly in high-volume settings. While these potential advantages require further validation in clinical studies, the present findings support the feasibility of integrating shorter equilibration approaches into existing vitrification protocols.

This study has several strengths. It prospectively evaluated the feasibility of shortened-equilibration vitrification across multiple developmental stages, including zygotes, cleavage-stage embryos, and blastocysts, within a single experimental framework. The use of a standardized warming protocol allowed consistent assessment of post-warming outcomes, minimizing variability related to procedural differences. In addition, the inclusion of multiple embryological endpoints, including survival, developmental progression, and blastocyst re-expansion, provides a comprehensive evaluation of the potential impact of shortened equilibration protocols.

This study has several limitations. The sample size was relatively limited, particularly within subgroup analyses, and the study may therefore be underpowered to detect clinically meaningful differences. The findings should therefore be interpreted as preliminary. As a proof-of-concept study, clinical outcomes such as implantation and live birth rates were not assessed. Ploidy status of blastocysts was not systematically assessed and therefore could not be controlled for in the analysis. For 3PN zygotes, two media systems (Sage and Kitazato) were used during the study period; however, internal comparisons in our laboratory did not demonstrate differences in survival or developmental outcomes between systems. In addition, the use of 3PN zygotes may not fully reflect the behavior of normally fertilized embryos. Nevertheless, the consistency of findings across different developmental stages and outcome measures supports the robustness of the observed effects and provides a basis for further investigation in larger, clinically oriented studies.

This proof-of-concept preclinical study was designed to evaluate embryological outcomes rather than clinical endpoints. Accordingly, clinical outcomes such as implantation, ongoing pregnancy, and live birth were not assessed. The present findings demonstrate the embryological feasibility of shortened-equilibration vitrification; evaluation of clinical safety and efficacy will require prospective clinical studies as a next step.

## Conclusion

In conclusion, shortened-equilibration vitrification with a markedly shortened equilibration step appears feasible across different developmental stages, without compromising post-warming survival or early embryological outcomes. These findings support the potential for simplifying vitrification protocols while maintaining biological integrity. Further studies are warranted to validate these results and assess clinical outcomes in routine practice.

## Supplementary Material

hoag053_Supplementary_Data

## Data Availability

The data underlying this article will be shared on reasonable request to the corresponding author.
