# Supplementary material for: Vitrification with shortened equilibration across human embryonic developmental stages: a proof-of-concept study
Source: Hum Reprod Open. 2026 Jun 3;2026(3):hoag053. doi: 10.1093/hropen/hoag053 (PMC13283424; doi:10.1093/hropen/hoag053)
Supplement: hoag053_Supplementary_Data [file hoag053_supplementary_data.pdf]

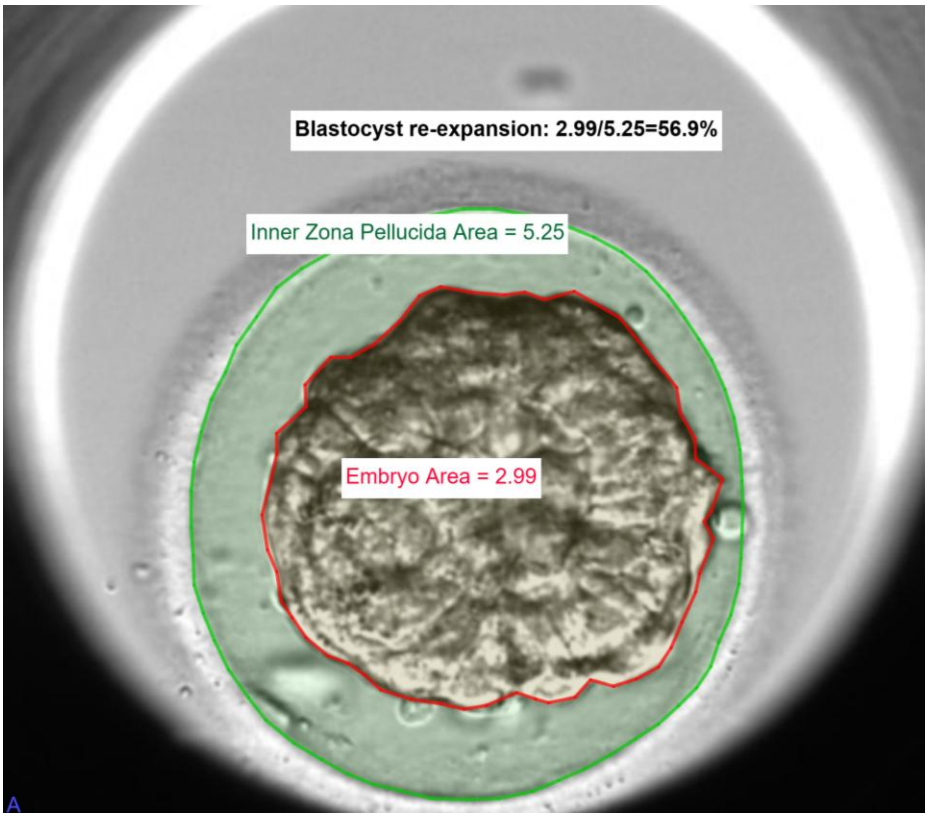

Supplementary Figure S1. Quantification and Monitoring of Blastocyst Re-expansion using geometric polygon analysis. The collapsed blastocyst margins were defined using the point tool to create the first polygon's area (red). The zona pellucida inner space margins were defined using the point tool to create the second polygon area (green). The area ratio was calculated by dividing the collapsed blastocyst area by the zona pellucida inner space area to determine the rate of re-expansion (56.9%).
